# Single-marker and multi-marker approaches to appraise the relationships between biomarkers and microalbuminuria in Chinese middle-aged and elderly from communities: a cross-sectional analysis

**DOI:** 10.1186/s12882-018-0888-3

**Published:** 2018-04-23

**Authors:** Shihui Fu, Yi Guo, Zhao Zhang, Leiming Luo, Ping Ye

**Affiliations:** 1 Department of Geriatric Cardiology, Chinese People’s Liberation Army General Hospital, Beijing, China; 2 Department of Cardiology and Hainan Branch, Chinese People’s Liberation Army General Hospital, Beijing, China

**Keywords:** Biomarkers, Communities, Microalbuminuria, Middle-aged and elderly

## Abstract

**Background:**

Analyzing the relationships between biomarkers representing distinct pathophysiologic pathways and microalbuminuria (MA) can strengthen the identifying ability for renal damage and illuminate previously unrecognized pathways for the pathogenesis of renal damage. The current analysis was to clarify the associations between biomarkers, including N-terminal prohormone of brain natriuretic peptide (NT-proBNP), high-sensitivity C-reactive protein (hsCRP), homocysteine and uric acid (UA), and MA in Chinese middle-aged and elderly from communities.

**Methods:**

All 839 residents had complete set of these biomarkers and full assessment of MA.

**Results:**

Prevalence of participants with MA was 13.5% (113 participants). Levels of age, systolic blood pressure (SBP), fasting blood glucose (FBG), homocysteine and NT-proBNP and proportion of cigarette smoking in participants with MA significantly exceeded those in participants without MA (*p* < 0.05 for all). In single-marker and multi-marker models of linear and logistic regression analyses, homocysteine and NT-proBNP levels (*p* < 0.05 for all) rather than hsCRP and UA levels (*p* > 0.05 for all) were statistically significant in relation to MA. Additionally, no matter which biomarker was directed at, levels of age, SBP and FBG and proportion of cigarette smoking had significant associations with MA. Homocysteine and NT-proBNP levels (*p* < 0.05 for all) rather than hsCRP and UA levels (*p* > 0.05 for all) had significant abilities to identify MA.

**Conclusion:**

Both single-marker and multi-marker analyses confirmed that homocysteine and NT-proBNP were associated with MA in Chinese middle-aged and elderly from communities after adjustment for multiple confounders.

## Background

Renal damage has significant associations with stroke, cardiovascular disease, peripheral vascular disease and all-cause mortality [1–3]. Microalbuminuria (MA) is well known as an early sign of kidney damage, and early screening of MA is significant to identify renal damage [4]. Analyzing the relationships between MA and biomarkers representing distinct biologic can strengthen our identifying ability for renal damage at early stage [5]. Previous studies relating biomarkers to MA have conducted mainly among diabetic and hypertensive individuals, and most of their samples were white, limiting the generalizability to other ethnicities [6–8]. Therefore, scarce studies have examined this kind of relationships in Chinese community population. The current analysis aimed to clarify the associations between biomarkers and MA in Chinese middle-aged and elderly from communities. What is more, the current analysis constructed single-marker and multi-marker models of linear and logistic regression analyses simultaneously and tried to test whether these associations were independent out of multiple potential confounders.

## Methods

### Study population

For the current analysis, 841 participants aged 45 years and over were included from a large health check-up survey, which began in May 2007 and ended in July 2009. A stratified cluster sampling was applied, and three districts (Fengtai, Shijingshan and Daxing) were chosen from eighteen districts in Beijing. Four communities were then chosen from these districts, and participants were finally chosen from these communities. After 2 participants with macroalbuminuria excluded, 839 participants attended the final analysis. All participants in the current analysis had no missing values for any covariates.

### Physical examination

Physical examination was performed by full trained physicians. Height and weight were measured, and body mass index (BMI) was defined as weight (kg) divided by height (m) squared. Participants with systolic blood pressure (SBP) ≥ 140 mmHg, diastolic blood pressure (DBP) ≥ 90 mmHg or hypotensive drugs were regarded to have hypertension. Mercury sphygmomanometer was applied to measure blood pressure twice and obtain an average value (Yuwell medical equipment & supply Co., Ltd., Jiangsu, China). Two measures are significantly related to each other (SBP: *r* = 0.925, *p* < 0.001; DBP: *r* = 0.908, *p* < 0.001). Cigarette smoking was defined as the consumption of one or more cigarettes per day for at least one recent year.

### Laboratory test

Fasting blood specimen was routinely collected between 8:00 AM and 10:00 AM, and stored at 4 °C until analyzed by the central laboratory on the same day. N-terminal prohormone of brain natriuretic peptide (NT-proBNP) (representing natriuretic peptide system; measured by electrochemiluminescence immunoassays [Roche Products Ltd., Basel, Switzerland] with an autoanalyzer [COBAS 6000; Roche Products Ltd., Basel, Switzerland]); high-sensitivity C-reactive protein (hsCRP) (representing systemic inflammation; measured by immunoturbidimetric assays with a Dimension RxL Max analyzer [Siemens Healthcare Diagnostics Inc., Munich, Germany]); homocysteine (representing oxidative stress; measured by high-performance chromatography with fluorometric detection); and uric acid (UA) (representing nucleic acid metabolism; measured by enzymatic assays [Roche Products Ltd., Basel, Switzerland] with an autoanalyzer [COBAS 6000; Roche Products Ltd., Basel, Switzerland]). Additionally, concentrations of fasting blood glucose (FBG), triglyceride (TG), high-density lipoprotein cholesterol (HDL-c) and low-density lipoprotein cholesterol (LDL-c) were quantified by enzymatic assays (Roche Products Ltd., Basel, Switzerland) with an autoanalyzer (Roche Products Ltd., Basel, Switzerland). Type 2 diabetes referred to those with FBG ≥ 7.0 mmol/L or hypoglycemic treatment. Concentrations of serum creatinine were measured by enzymatic assays (Roche Products Ltd., Basel, Switzerland) with a Hitachi 7600 autoanalyzer (Hitachi, Tokyo, Japan). Estimated glomerular filtration rate (eGFR) was calculated with Chinese modified Modification of Diet in Renal Disease equation: 175 × serum creatinine (mg/dl)^-1.234^ × age (year)^-0.179^ × 0.79 (if female) [9]. Urinary albumin (UMA) was measured on at least 5 mL of midstream urine from morning urine. Participants were categorized as those without MA and macroalbuminuria (< 20 mg/L), those with MA (20–199 mg/L) and those with macroalbuminuria (≥ 200 mg/L).

### Statistical analysis

Statistical analysis was undertaken by Statistical Package for Social Science version 17 software (SPSS Inc., Chicago, IL, USA). Categorical variable was described with number and proportion. Continuous variable (normal distribution) was described with mean and standard deviation. Continuous variable (skewed distribution) was described with median and interquartile range. Student’s t-test (continuous variable with normal distribution), Mann–Whitney U test (continuous variable with skewed distribution) and Chi-squared test (categorical variable) were performed to compare differences between participants with and without MA. In order to analyze the independent relationships between biomarkers and MA, essential variable was log-transformed in accordance with multinormality assumption, and multiple linear [independent variable: biomarkers; dependent variable: UMA] and logistic regression models (independent variable: biomarkers; dependent variable: MA) were evaluated following three steps: 1) step 1 included single biomarker in the model (covariates: age and gender); 2) step 2 included single biomarker in the model (covariates: age, gender, BMI, cigarette smoking, SBP, triglyceride, HDL-c, LDL-c, FBG and eGFR); and 3) step 3 included all biomarkers simultaneously in the model (covariates: age, gender, BMI, cigarette smoking, SBP, triglyceride, HDL-c, LDL-c, FBG and eGFR). Statistical significance was deemed as two-tailed *P* < 0.05.

## Results

### Baseline characteristics

Age ranged from 48 to 89 years, with a median of 67 years; 34.4% were males, 25.3% had cigarette smoking, 59.7% had hypertension, and 25.0% had type 2 diabetes. Prevalence of participants with MA was 13.5% (113 participants). Table 1 compared the characteristics of study population according to presence versus absence of MA, and levels of age, BMI, SBP, DBP, FBG, homocysteine and NT-proBNP and proportions of cigarette smoking, hypertension and angiotensin converting enzyme inhibitor/angiotensin receptor blocker treatment in participants with MA significantly exceeded those in participants without MA.

**Table 1:** Characteristics of study population

Characteristics	ALL	NA	MA	*P* value
	(*n* = 839)	(*n* = 726)	(*n* = 113)	
Age (year)	67 (61–72)	62 (56–69)	67 (62–72)	< 0.001
Males (%)	289 (34.4)	246 (33.9)	43 (38.1)	0.386
BMI (kg/m^2^)	25.26 (23.11–27.64)	25.11 (22.96–27.62)	26.22 (24.29–27.70)	0.009
Cigarette smoking (%)	212 (25.3)	169 (23.3)	43 (38.1)	0.001
Hypertension (%)	501 (59.7)	417 (57.4)	84 (74.3)	0.001
Type 2 diabetes (%)	210 (25.0)	182 (25.1)	28 (24.8)	0.947
SBP (mmHg)	130 (120–144)	130 (120–142)	138 (123–157)	< 0.001
DBP (mmHg)	77 (70–83)	75 (70–81)	80 (75–90)	< 0.001
Triglyceride (mmol/L)	1.53 (1.16–2.15)	1.53 (1.15–2.12)	1.54 (1.21–2.40)	0.350
HDL-c (mmol/L)	1.34 (1.10–1.59)	1.33 (1.09–1.60)	1.36 (1.15–1.54)	0.695
LDL-c (mmol/L)	3.01 (2.55–3.43)	3.02 (2.56–3.42)	2.95 (2.52–3.48)	0.803
FBG (mmol/L)	5.01 (4.35–5.79)	4.88 (4.30–5.70)	5.48 (4.92–6.39)	< 0.001
eGFR (ml/min/1.73 m^2^)	86.82 (78.09–97.65)	86.61 (78.13–97.57)	89.09 (77.54–98.47)	0.972
UMA (mg/L)	1.39 (0.61–6.35)	1.12 (0.55–2.93)	41.04 (28.18–94.35)	< 0.001
UA (μmol/L)	288.30 (241.80–343.00)	288.60 (241.30–341.28)	286.10 (244.65–348.05)	0.455
Homocysteine (μmol/L)	18.30 (15.30–22.40)	17.90 (15.10–21.63)	20.70 (16.85–24.35)	< 0.001
HsCRP (mg/dl)	0.21 (0.13–0.35)	0.21 (0.13–0.35)	0.21 (0.11–0.34)	0.107
NT-proBNP (pg/mL)	47.60 (23.20–90.00)	45.20 (21.75–85.90)	73.75 (36.94–132.10)	< 0.001
ACEI/ARBs (%)	34 (4.1)	23 (3.2)	11 (9.7)	0.002
Statins (%)	32 (3.8)	31 (4.3)	1 (0.9)	0.138

Abbreviation: *MA* microalbuminuria, *BMI* body mass index, *SBP* systolic blood pressure, *DBP* diastolic blood pressure, *HDL-c* high-density lipoprotein-cholesterol, *LDL-c* low-density lipoprotein-cholesterol, *FBG* fasting blood glucose, *eGFR* estimated glomerular filtration rate, *UMA* urinary albumin, *UA* uric acid, *hsCRP* high-sensitivity C-reactive protein, *NT-proBNP* N-terminal prohormone of brain natriuretic peptide, *ACEI/ARB* angiotensin converting enzyme inhibitors/angiotensin receptor blockers

### Single-marker analysis

In the first and second steps of linear (Table 2) and logistic (Table 3) regression models, homocysteine and NT-proBNP levels (*p* < 0.05 for all) rather than hsCRP and UA levels (*p* > 0.05 for all) were statistically significant in relation to UMA or MA. Additionally, no matter which biomarker was directed at in the first and second steps, levels of age, SBP and FBG and proportion of cigarette smoking had significant associations with MA (*p* < 0.05 for all).

**Table 2:** Single-marker and multi-marker analyses in the linear regression

Biomarkers	Models	UMA	
		Standardized β value	*P* value
Homocysteine (μmol/L)	1st step^a^	0.275	< 0.001
	2nd step^b^	0.236	< 0.001
	3rd step^c^	0.223	< 0.001
HsCRP (mg/dl)	1st step^a^	−0.018	0.590
	2nd step^b^	−0.005	0.888
	3rd step^c^	−0.002	0.943
NT-proBNP (pg/mL)	1st step^a^	0.152	< 0.001
	2nd step^b^	0.140	< 0.001
	3rd step^c^	0.129	< 0.001
UA (μmol/L)	1st step^a^	0.001	0.987
	2nd step^b^	−0.049	0.199
	3rd step^c^	−0.054	0.148

Note: ^a^First step: single biomarker in the linear regression model adjusted by age and gender; ^b^Second step: single biomarker in the linear regression model adjusted by age, gender, body mass index, cigarette smoking, systolic blood pressure, triglyceride, high-density lipoprotein-cholesterol, low-density lipoprotein-cholesterol, fasting blood glucose and glomerular filtration rate; ^c^Third step: all biomarkers simultaneously in the linear regression model adjusted by age, gender, body mass index, cigarette smoking, systolic blood pressure, triglyceride, high-density lipoprotein-cholesterol, low-density lipoprotein-cholesterol, fasting blood glucose and glomerular filtration rate

Abbreviation: *UMA* urinary albumin, *hsCRP* high-sensitivity C-reactive protein, *NT-proBNP* N-terminal prohormone of brain natriuretic peptide, UA: uric acid

**Table 3:** Single-marker and multi-marker analyses in the logistic regression

Biomarkers	Models	MA	
		OR value (95% CI)	*P* value
Homocysteine (μmol/L)	1st step^a^	1.066 (1.041–1.092)	< 0.001
	2nd step^b^	1.059 (1.031–1.087)	< 0.001
	3rd step^c^	1.059 (1.032–1.087)	< 0.001
HsCRP (mg/dl)	1st step^a^	0.742 (0.426–1.293)	0.293
	2nd step^b^	0.544 (0.256–1.160)	0.115
	3rd step^c^	0.490 (0.218–1.098)	0.083
NT-proBNP (pg/mL)	1st step^a^	1.002 (1.001–1.003)	0.006
	2nd step^b^	1.002 (1.000–1.003)	0.020
	3rd step^c^	1.001 (1.000–1.003)	0.043
UA (μmol/L)	1st step^a^	1.002 (0.999–1.005)	0.111
	2nd step^b^	1.001 (0.998–1.005)	0.415
	3rd step^c^	1.001 (0.998–1.005)	0.478

Note: ^a^First step: single biomarker in the logistic regression model adjusted by age and gender; ^b^Second step: single biomarker in the logistic regression model adjusted by age, gender, body mass index, cigarette smoking, systolic blood pressure, triglyceride, high-density lipoprotein-cholesterol, low-density lipoprotein-cholesterol, fasting blood glucose and glomerular filtration rate; ^c^Third step: all biomarkers simultaneously in the logistic regression model adjusted by age, gender, body mass index, cigarette smoking, systolic blood pressure, triglyceride, high-density lipoprotein-cholesterol, low-density lipoprotein-cholesterol, fasting blood glucose and glomerular filtration rate

Abbreviation: *MA* microalbuminuria, *OR* odd ratio, *CI* confidence interval, *hsCRP* high-sensitivity C-reactive protein, *NT-proBNP* N-terminal prohormone of brain natriuretic peptide, *UA* uric acid

### Multi-marker analysis

**Fig. 1:**
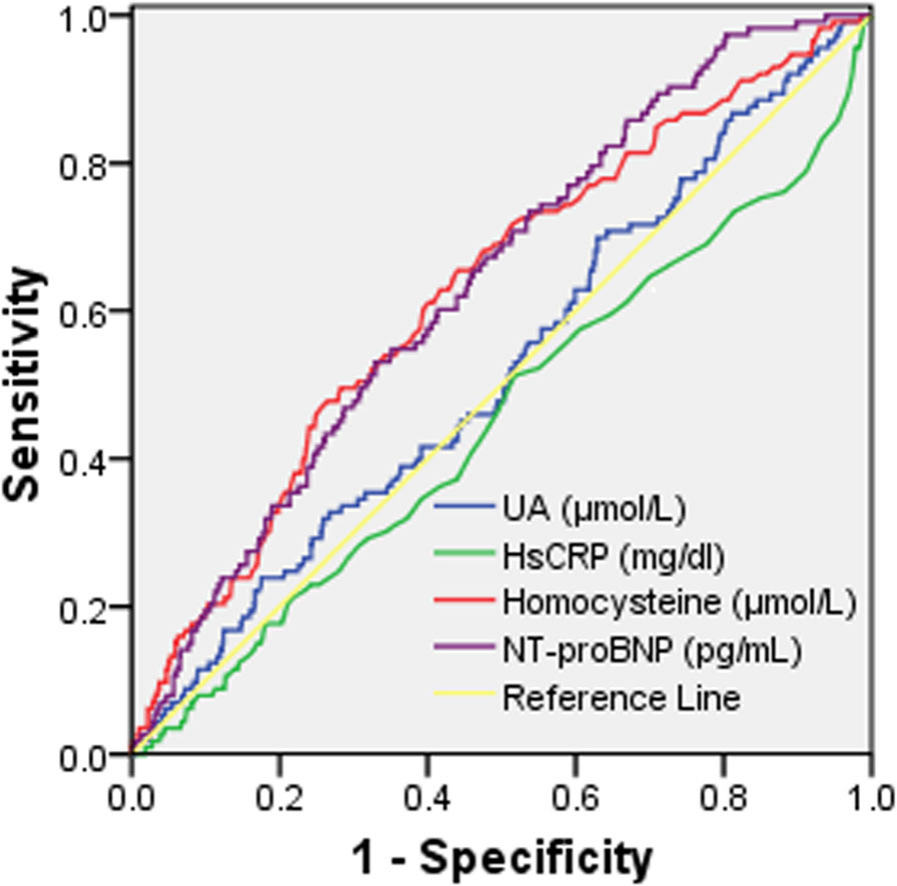
Receiver Operator Characteristic Curve for abilities of biomarkers to identify MA. Abbreviation: *hsCRP* high-sensitivity C-reactive protein, *NT-proBNP* N-terminal prohormone of brain natriuretic peptide, *UA* uric acid

In the third steps of linear (Table 2) and logistic (Table 3) regression models, homocysteine and NT-proBNP levels (*p* < 0.05 for all) rather than hsCRP and UA levels (*p* > 0.05 for all) were statistically significant in relation to UMA or MA. Additionally, no matter which biomarker was directed at in the third steps, levels of age, SBP and FBG and proportion of cigarette smoking had significant associations with MA. Homocysteine and NT-proBNP levels (*p* < 0.05 for all) rather than hsCRP and UA levels (*p* > 0.05 for all) had significant abilities to identify MA (Table 4 and Fig. 1).

**Table 4:** Abilities of biomarkers to identify MA

	AUC value (95% CI)	*P* value
Homocysteine (μmol/L)	0.627 (0.572–0.682)	< 0.001
HsCRP (mg/dl)	0.453 (0.393–0.513)	0.108
NT-proBNP (pg/mL)	0.636 (0.585–0.687)	< 0.001
UA (μmol/L)	0.522 (0.465–0.579)	0.455

Abbreviation: *MA* microalbuminuria, *AUC* area under the curve, *CI* confidence interval, *hsCRP* high-sensitivity C-reactive protein, *NT-proBNP* N-terminal prohormone of brain natriuretic peptide, *UA* uric acid

## Discussion

In this community population, we observed a significant relationships of homocysteine and NT-proBNP with MA even after multivariate adjustment. These data suggested that homocysteine and NT-proBNP representing different pathophysiologic pathways can promote the identification of individuals with increased risk for developing renal damage. In addition, these findings demonstrated several pathophysiologic pathways playing a potential role in the pathogenesis of renal damage.

Produced by the demethylation of methionine, homocysteine has been considered to have significant association with MA in some studies [10, 11], but not in other study [12]. This analysis extended the current literature and reported that increased homocysteine levels were independently associated with MA in Chinese community population [13]. The pathophysiologic pathways between hyperhomocysteinemia and MA remain unclear. Previous studies have suggested that homocysteine-related oxidative stress injures endothelial and mesangial cells [14]. Renal endothelial and mesangial cells are significant for not only controlling intraglomerular pressure, but also preserving glomerular size and charge selectivity. If they are injured, there will be elevated intraglomerular pressure, and reduced glomerular charge and size selectivity, both causing MA. Additionally, there are other common pathophysiologic pathways, such as vitamin B6, B12 and folate deficiency, resulting in both hyperhomocysteinemia and MA [15].

Meanwhile, this analysis identified NT-proBNP as a biomarker associated with MA. Previous study has suggested that NT-proBNP have significant association with renal damage [8]. Because NT-proBNP is removed by kidney, early reduction of NT-proBNP clearance can account for our finding [16]. Besides, natriuretic peptides can prevent glomerular hyperfiltration, fibrosis and hypertrophy in previous experiments, so NT-proBNP may have protective effect on kidney [17–19].

The current analysis had strength and limitations. The current analysis specially aimed at Chinese middle-aged and elderly from communities, and had a large number of participants without missing values for any covariates. Moreover, it clarified the associations between biomarkers and MA through constructing single-marker and multi-marker models of linear and logistic regression analyses simultaneously. But UMA was evaluated on at least 5 mL of midstream urine from morning urine rather than 24-h urine. It is difficult and unpractical to measure 24-h urine in epidemiological investigations.

## Conclusion

Both single-marker and multi-marker analyses provided proof that homocysteine and NT-proBNP levels were significantly associated with MA in Chinese middle-aged and elderly from communities after adjustment for multiple confounders. These biomarkers representing different pathophysiologic pathways can enhance the identification of individuals with increased risk for developing renal damage. More importantly, these findings can help us understand these potential pathways involved in the pathogenesis of renal damage.
